# The concordance between IHC and ISH for HER-2 testing in breast cancer in Nakhon Pathom Hospital, Thailand, based on the ASCO/CAP 2018 guidelines: a retrospective study

**DOI:** 10.3332/ecancer.2022.1370

**Published:** 2022-03-31

**Authors:** Tawasapon Thambamroong

**Affiliations:** Medical Oncology Unit, Department of Medicine, Nakhon Pathom Hospital, Nakhon Pathom 73000, Thailand

**Keywords:** HER-2 breast cancer, ISH, IHC, FISH, dual-probe ISH, breast cancer, Thailand, ASCO/CAP 2018

## Abstract

Human epidermal growth factor receptor 2 (HER-2) is the prognostic and predictive biomarker for breast cancer found in a quarter of all breast cancer cases. Precise testing of HER-2 will impact treatment response.Based on the updated American Society of Clinical Oncology/College of American Pathologists (ASCO/CAP) HER-2 testing guidelines, we report 141 cases of positive concordance rate between HER-2 IHC stain and dual-probe ISH for HER-2 testing classified in equivocal (2+) and positive (3+) IHC groups in Nakhon Pathom Hospital, Thailand. Our study showed statistical significance in the relationship between positive (3+) IHC and dual-probe ISH for HER-2 testing with a correlation rate of 91.76% (*r*_s_ = 0.38031, *p* < 0.0001) and 37.50% in equivocal (2+) IHC groups.This is the first report of a positive concordance rate between equivocal and positive HER-2 IHC and dual-probe ISH for HER-2 based on the ASCO/CAP 2018 guidelines in Thailand. Our study confirmed a statistically significant relationship in the positive IHC (3+) group. In addition, we suggested reflexing ISH testing only in equivocal IHC (2+) cases to decrease the waiting time and economical approach.

## Background

Breast cancer is the most prevalent cancer among females in the world. Furthermore, it causes the highest mortality rate. In Thailand, the cancer registry database from the National Cancer Institute from 2016 to 2018 showed an age-standardised rate (ASR) of 34.2:100,000 female population. Similar to Nakhon Pathom, in the west suburban area of Bangkok, Thailand, a 22.5:100,000 female population ASR average was found from 2016 to 2020, and is increasing.

Human epidermal growth factor receptor 2 (HER-2)-positive breast cancer comprises 20%–30% breast cancers caused by an abnormality of the HER-2 gene on the 17th chromosome, resulting in amplification and overexpression of the cancer cell surface field [1]. The HER-2 gene is the prognostic factor to breast cancer survival and the predictive factor to the treatment response of anti-HER-2 drugs. Various studies have reported the significant benefits of anti-HER-2 agents, improving relapse-free survival in the early stage [2–5] and decreasing the mortality rate in advanced stage breast cancer [6, 7]. Furthermore, these benefits have been based on precise HER-2 testing.

The American Society of Clinical Oncology (ASCO) and the College of American Pathologists (CAP) agree on the HER-2 testing recommendations, updated in 2018. They recommended the immunohistochemistry stain (IHC) for HER-2 as the standard of care. In the IHC for HER-2 testing, they proposed incomplete membrane staining as faint or barely perceptible with more than 10% of the tumour cells as IHC 1+. No staining or incomplete, faint or barely perceptible membrane staining with less than or equal to 10% of the tumour cells was IHC 0, and both were classified as ‘IHC negative’. They also updated the report of ‘positive IHC staining’ or 3+ to complete cell membrane staining of more than 10% of the overall cancer cells. The partial cell membrane IHC stains more than 10% of cancer cells for ‘equivocal’ or 2+, and recommends further investigation with *in situ* hybridisation (ISH). Fluorescence *in situ* hybridisations (FISH) is the gold standard or alternative to dual *in situ* hybridisation to confirm the diagnosis on the same specimen, or when a new specimen is available to perform IHC, and later ISH, if necessary [8].

In HER-2 IHC equivocal or 2+, the ASCO/CAP 2018 guidelines recommend reflex ISH testing on the same tissue or to order a new specimen test when available with both IHC and ISH. ISH reports will be categorised in five groups based on the HER-2 clones. Group 1 constitutes classic HER-2 amplification: single-probe average HER2 copy number ≥6.0 signals/cell or dual-probe HER2/CEP17 ratio of ≥2.0, with an average HER2 copy number ≥4.0 signals/cell and will be reported as positive. Conversely, Group 5 comprises classic HER-2 non-amplification, including the single-probe average HER2 copy number <4.0 signals/cell or dual-probe HER2/CEP17 ratio of <2.0, with an average HER2 copy number of 4.0 signals/cell and will be reported as negative. Group 2 or monosomy chromosome 17 involves dual-probe HER-2/CEP17 ratio ≥2.0, with an average HER2 copy number <4 signals/cell. Group 4 or borderline HER-2 amplification includes dual-probe HER-2/CEP17 ratio <2.0, with an average HER2 copy number ≥4 and <6 signals/cell and will be reported positive if concurrent IHC is 3+. If not, further dual-probe ISH or additional workup is required. Lastly, in group 3 or co-amplification, previously polysomy chromosome 17, the dual-probe ISH HER-2/CEP17 ratio <2.0 with an average HER-2 copy number ≥6 signals/cell will be reported positive if concurrent IHC 3+ or concurrent IHC 2+ with observer, blinded to previous results recounts ISH, at least 20 cells, and in concordance with the new result [8] (Table 1).

For equivocal results on single-probe ISH, the dual-probe ISH needs to be carried out for the final result [8].

Many studies have carried out the significant positive result improvement of IHC stain, compared with ISH [9, 10] based on the updated ASCO/CAP 2018 guidelines. However, in Thailand, the confirmation of the ISH test is recommended for all equivocal (2+) or positive (3+) IHC testing because of reimbursement issues.

This retrospective study aimed to demonstrate the positive concordance rate between IHC and FISH for HER-2 testing in breast cancer based on the ASCO/CAP 2018 recommendations to confirm positive IHC results for reimbursed anti-HER-2 drugs and decrease the test waiting time to benefit patients in Thailand.

## Methods

### Study population

**Figure 1.**
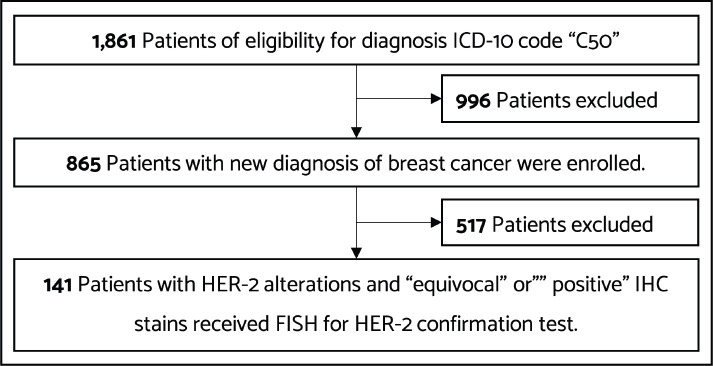
Flow diagram showing patients included in the study.

This single-institution retrospective study enrolled female patients who were newly diagnosed with breast cancer with HER-2 alteration at Nakhon Pathom Hospital between 1 January 2020 and 30 October 2021. We had changed to the ASCO/CAP 2018 recommendations. Eligible patients comprised adult females with histologically confirmed invasive ductal carcinoma (IDC) with IHC stain results of ‘equivocal (2+)’ or ‘positive (3+)’ confirmed with dual-probe ISH for HER-2 testing. We searched for patients with C50 International Classification of Diseases (ICD) -10 code in our hospital database. Of 1,861 patients with the C50 ICD-10 code, 865 patients received a new diagnosis of breast cancer. Patients who had negative results for HER-2 and did not confirm dual-probe ISH for HER-2 testing were excluded. We reviewed the data of 141 patients who had equivocal or positive IHC stain results, confirmed with dual-probe ISH for HER-2 testing. The flow diagram of the patients included in the study is shown in Figure 1.

### Dual-probe FISH

The FISH assay was carried out on formalin-fixed, paraffin-embedded specimens using the Path-Vyson HER-2 DNA Probe Kit from Abbott Laboratories. All cases used a dual probe (HER-2 and CEP17). The interpretation of results was based on the 2018 ASCO/CAP guidelines.

### Immunohistochemistry

IHC staining was carried out on freshly cut formalin-fixed, paraffin-embedded sections of 5 µm from the same specimen blocks that were used for testing the HER-2 dual-probe ISH using PATHWAY anti-HER-2/neu (4B5) rabbit monoclonal antibody from Ventana Medical Systems, Inc. The interpretation of results was based on the 2018 ASCO/CAP guidelines.

We recorded patient characteristics such as age, tumour size, lymph node involvement, metastatic size, tumour staging based on the 8th AJCC classification, hormonal status, HER-2 status and Ki-67 at diagnosis. The data cut-off date was 30 October 2021.

### Statistical analysis

The primary endpoint was the concordance rate between positive (3+) HER-2 IHC stain and FISH for HER-2 testing in Nakhon Pathom Hospital. The secondary endpoint was the concordance rate between equivocal (2+) HER-2 IHC stain and FISH for HER-2. We used descriptive statistics to identify baseline clinical characteristics of HER-2 alterations in breast cancer. The results were shown in correlation with percentage and then analysed using Spearman’s rank-order correlation.

## Results

Between January 2020 and October 2021, we recorded 1,861 patients who received a diagnosis of breast cancer. From 1,861 patients, 865 had new diagnoses. Furthermore, 141 patients exhibited HER-2 equivocal or positive IHC staining and confirmed dual-probe ISH studies were included in this study.

### Demographic data

Of the 141 patients with HER-2 breast cancer, all were women, and the mean age was 55 ± 11.88 (SD) years. Most presented tumours more extensive than 20 mm but smaller than 50 mm (72/141; 51%), and 25.3% (36/141) had localised invasions. Nearly one-half exhibited 1–3 regional lymph nodes metastases, and only 12 patients (8.5%) had distant metastases. Two-thirds of the patients had high oestrogen receptor expression. The median Ki-67 expression was 42.62 ± 18.80%. In subgroups characterised by HER-2-IHC stain, the HER-2-positive or IHC 3+ indicated a more aggressive disease than HER-2 equivocal or IHC 2+. The baseline clinical characteristics of HER-2 alterations among female patients with breast cancer are shown in Table 2.

In the baseline genomic characteristics of HER-2 alteration, this study shows that most IHC-positive or 3+ patients have classical HER-2 amplification by dual-probe ISH based on the ASCO/CAP 2018 guidelines. However, in the IHC equivocal or 2+ group, there share of varieties of HER-2 clones may interfere with the result of the IHC (Table 3).

### Concordance rate between HER-2 IHC and ISH

The patients were categorised into two groups depending on HER-2 IHC staining statuses. Fifty-six patients were categorised in the equivocal (2+) HER-2 IHC staining group and 85 patients were categorised in the positive (3+) HER-2 IHC stain group. All patients had confirmation ISH testing.

### Equivocal HER-2 IHC group

Altogether, 56 patients comprised this group. Thirty-two patients revealed negative results on ISH testing and 21 patients were confirmed positive. Furthermore, three patients reported equivocal and repeated observer, blinded to previous results, confirming negative ISH. The correlation was 37.5%, without statistical significance.

### Positive HER-2 IHC group

Totally, 85 patients comprised this group. Almost all patients were confirmed ISH-positive; only seven were negative and no equivocal result was noted. The correlation was 91.76%, with Spearman’s rank-order correlation being 0.38031 and a statistically significant *p*-value (Table 4).

## Discussion

The ASCO/CAP guidelines for HER-2 reporting in breast cancer were first published in 2007 and updated in 2013. The 2018 ASCO/CAP guidelines constitute the latest version. Compared with the 2013 ASCO/CAP guidelines, the 2018 ASCO/CAP guidelines refined the HER-2 evaluation algorithms by defining uncommon HER-2 ISH amplification patterns that cause uncertain biological and clinical significance. They increased the emphasis on coordinating ISH and IHC results [11], refining the criteria for HER-2-positive patients with monosomy chromosome 17 and co-amplification of HER-2 incorporation with HER-2 protein expression by IHC. These resulted in significantly decreased equivocal results and increased truly negative results in HER-2 IHC stains [9, 12].

These guidelines recommend reporting that HER-2 IHC 3+ is HER-2-positive. However, in Thailand, we need to carry out reflex ISH testing for HER-2 to confirm all IHC 3+ only for the proposed reimbursement, leading to delays in therapeutic decisions and increasing the financial burden for reflex testing. In related studies, Gordian-Arroyo *et al* [12] showed that all patients with HER-2 IHC 3+ were positive for FISH for HER-2, of which 97.8% (134/137) were classic amplified HER-2 or dual-probe ISH presenting a HER2/CEP17 ratio of ≥2.0, with an average HER2 copy number ≥4.0 signals/cell or group 1. In our study, 91.76% (78/85) of HER-2 IHC 3+ with reflex ISH-positive were categorised as classical HER-2 amplification or dual-probe ISH group 1. Spearman’s rank-order correlation was 0.38031 and statistically significant. These could ensure that all patients with HER-2 IHC 3+ stain, based on the ASCO/CAP 2018 recommendations, could be interpreted as HER-2-positive, which was not mandatory for reflex ISH testing with HER-2 dual-probe.

However, in the equivocal HER-2 IHC group, various results were found of dual-probe ISH. Most were classified as classical HER-2 non-amplification clones or negative FISH (29/56; 51.8%); others were classical HER-2 amplification (21/56; 37.5%), co-amplification (3/56; 5.4%), borderline HER-2 amplification (2/56; 3.5%) and monosomy chromosome 17 (1/56; 1.8%), respectively (Table 3). In a related study, the proportion of dual-probe ISH HER-2 clones varied but was also predominant in classic HER-2 non-amplification and classic HER-2 amplification (Table 5). This diversified dual-probe ISH group resulted in the heterogeneity of HER-2 alteration, affecting HER-2 protein expression on the cell surface, causing partial cell membrane staining to result in equivocal (2+) IHC. This explained why the 2018 ASCO/CAP guidelines suggest interpreting results concurrent with IHC status. In addition, it may involve recounting ISH with an observer, blinded to previous results in monosomy chromosome 17, co-amplification or borderline HER-2 amplification groups.

From the results, we suggest that the reflex ISH testing for all HER-2 IHC-positive or 3+ patients is unnecessary, which can decrease the waiting time and financial burden of those receiving the same treatment benefits from anti-HER-2 drugs, in both adjuvant and palliative settings [3–7]. Acceptance of the equivocal cases is subjected to reflex ISH testing for the final result.

Our study encountered several limitations. It constituted a single-centre retrospective study. The sample size was too small to confirm dual-probe ISH testing results. Furthermore, clinical outcomes could not be determined due to the sample size and short follow-up time.

## Conclusion

This study constitutes the first report of a positive concordance rate between equivocal and positive HER-2 IHC and FISH for HER-2, based on the ASCO/CAP 2018 guidelines in Thailand. Our study confirmed the statistically significant relationship in the positive IHC (3+) group. In addition, we suggest reflex ISH testing only in equivocal IHC (2+) cases to decrease waiting time and expense.

## Conflicts of interest

The author declares no conflicts of interest.

## Author’s contributions

Conceived and designed the analysis: TT; collected data: TT; contributed data or analysis tools: TT; performed the analysis: TT; and wrote the paper: TT.

## Ethical approval

Ethics approval was obtained by the Nakhon Pathom Hospital Ethics Committee.

## Funding

This research was fully funded by Nakhon Pathom Hospital, Thai Ministry of Public Health.

## Figures and Tables

**Table 1.** 2018 ASCO/CAP guidelines for HER-2 dual-probe ISH clinical subgroups and final results determination based on the integration with IHC results.

Group	HER-2 clones	Dual-probe ISH results		IHC	2018 ASCO/CAP recommendation
		HER2/CEP17	Average HER-2 copy number		
1	Classic HER-2 amplification	≥2.0	≥4	−	Positive
2	Monosomy 17	≥2.0	<4	3+	Positive
3	Co-amplification (previously polysomy 17)	<2.0	≥6	2+* or 3+	Positive
4	Borderline HER-2 amplification	<2.0	≥4 and <6	3+	Positive
5	Classic HER-2 non-amplification	<2.0	<4	−	Negative

* if IHC is 2+, the observer, blinded to previous results recount ISH, at least 20 cells, and the given concordance result will be classified as positive

**Table 2.** Clinical characteristics of HER-2 alterations among female patients with breast cancer at Nakhon Pathom Hospital, Thailand.

Characteristic		Total (*n* = 141)	HER-2 IHC Equivocal (2+) (*n* = 56)	HER-2 IHC Positive (3+) (*n* = 85)
Median age, years		55 ± 11.88	55 ± 11.02	55 ± 12.44
	Age <40 years	18 (12.8%)	9 (16.1%)	9 (10.6%)
	Age ≥40 years	123 (87.2%)	47 (83.9%)	76 (89.4%)
Tumour characteristics				
	Tumour ≤20 mm	6 (4.3%)	6 (10.7%)	0 (0%)
	Tumour >20 mm but ≤50 mm	72 (51.0%)	32 (57.1%)	40 (47.1%)
	Tumour >50 mm	27 (19.0%)	9 (16.1%)	18 (21.2%)
	Tumour with local invasion	36 (25.3%)	9 (16.1%)	27 (31.8%)
Nodal characteristics				
	No regional lymph node	15 (10.6%)	3 (5.4%)	12 (14.1%)
	1–3 nodal metastases	66 (46.8%)	29 (51.8%)	37 (43.5%)
	4–9 nodal metastases or ipsilateral internal mammary node metastases	24 (17.0%)	12 (21.4%)	12 (14.1%)
	>10 nodal metastases or level III axillary node or ipsilateral supraclavicular lymph node	36 (25.5%)	12 (21.4%)	24 (28.2%)
Distant metastasis				
	No distant metastasis	129 (91.5%)	50 (89.3%)	79 (92.9%)
	Distant metastasis	12 (8.5%)	6 (10.7%)	6 (7.1%)
Hormonal status				
	ER <10%	51 (36.2%)	18 (32.1%)	33 (38.8%)
	ER ≥10%	90 (63.8%)	38 (67.9%)	52 (61.2%)
Ki-67, %		42.62 ± 18.80	36.34 ± 18.49	42.26 ± 18.12

**Table 3.** Genomic HER-2 alteration clone characteristics by dual-probe ISH and IHC for HER-2 based on the 2018 ASCO/CAP guidelines among female patients with breast cancer at Nakhon Pathom Hospital, Thailand.

		HER-2 clone groups		n (%)
IHC	2+	1	Classic HER-2 amplification	21 (37.5)
		2	Monosomy 17	1 (1.8)
		3	Co-amplification (previously polysomy 17)	3 (5.4)
		4	Borderline HER-2 amplification	2 (3.5)
		5	Classic HER-2 non-amplification	29 (51.8)
	3+	1	Classic HER-2 amplification	78 (91.8)
		2	Monosomy 17	0 (0)
		3	Co-amplification (previously polysomy 17)	0 (0)
		4	Borderline HER-2 amplification	0 (0)
		5	Classic HER-2 non-amplification	7 (0)

**Table 4.** Correlation between IHC and dual-probe ISH for HER-2 based on the 2018 ASCO/CAP guidelines.

Testing modalities		Dual-probe ISH			Total	Correlation (%)
		Negative	Equivocal	Positive		
IHC	2+	32	3	21	56	37.50
	3+	7	0	78	85	91.76*
**Total**		36	3	99	141	

* *r_s_* = 0.38031, *p* < 0.0001

**Table 5.** Review of the comparison of dual-probe ISH results in equivocal IHC based on the 2018 ASCO/CAP guidelines.

No.	Author	FISH result, *n* (%)				
		Group 1	Group 2	Group 3	Group 4	Group 5
1.	Current author	21 (37.5)	1 (1.8)	3 (5.4)	2 (3.5)	29 (51.8)
2.	Gordian-Arroyo *et al* [12]	24 (10.0)	1 (0.4)	9 (3.7)	78 (32.5)	128 (53.3)
3.	Farshid *et al* [11]	39 (10.3)	3 (0.8)	2 (0.5)	22 (5.8)	313 (82.6)
4.	Pasricha *et al* [9]	35 (28.3)	3 (2.4)	3 (2.4)	4 (3.2)	79 (63.7)
5.	Murray *et al* [13]	106 (10.2)	75 (7.2)	1 (0.1)	38 (3.6)	824 (78.9)
6.	Li *et al* [14]	295 (13.1)	52 (2.3)	27 (1.2)	164 (0.1)	1716 (76.1)
